# Analysis of CLIP and iCLIP methods for nucleotide-resolution studies of protein-RNA interactions

**DOI:** 10.1186/gb-2012-13-8-r67

**Published:** 2012-08-03

**Authors:** Yoichiro Sugimoto, Julian König, Shobbir Hussain, Blaž Zupan, Tomaž Curk, Michaela Frye, Jernej Ule

**Affiliations:** 1 MRC Laboratory of Molecular Biology, Hills Road, Cambridge CB2 0QH, UK; 2 The Wellcome Trust Centre for Stem Cell Research, Tennis Court Road, Cambridge CB2 1QR, UK; 3 Faculty of Computer and Information Science, University of Ljubljana, Tržaška 25, SI-1000, Ljubljana, Slovenia

## Abstract

UV cross-linking and immunoprecipitation (CLIP) and individual-nucleotide resolution CLIP (iCLIP) are methods to study protein-RNA interactions in untreated cells and tissues. Here, we analyzed six published and two novel data sets to confirm that both methods identify protein-RNA cross-link sites, and to identify a slight uridine preference of UV-C-induced cross-linking. Comparing Nova CLIP and iCLIP data revealed that cDNA deletions have a preference for TTT motifs, whereas iCLIP cDNA truncations are more likely to identify clusters of YCAY motifs as the primary Nova binding sites. In conclusion, we demonstrate how each method impacts the analysis of protein-RNA binding specificity.

## Background

To understand post-transcriptional regulation, it is crucial to study protein-RNA interactions in the cellular environment. Irradiation with UV-C light creates a covalent bond between proteins and RNAs that are in direct contact *in vivo *without requiring pre-incubation of cells with photoreactive ribonucleoside analogs. Cross-linking and immunoprecipitation (CLIP) was therefore developed to identify RNA sites in direct contact with RNA-binding proteins (RBPs) [1]. Especially in combination with high-throughput sequencing, CLIP (or HITS-CLIP) identified RNA targets of RBPs in a transcriptome-wide manner [2-5]. These studies showed that the precise position of protein binding sites on target RNAs is extremely important, since the effect of RBPs on the alternative splicing largely depends on their precise binding position. This was most clearly shown by genome-wide RNA maps of splicing regulation [6,7].

To understand the precise position of protein-RNA cross-linking, several modifications of CLIP were developed. All of these approaches exploit the effect of cross-linked nucleotides during the reverse transcription reaction. One such approach, Photoactivatable Ribonucleoside-Enhanced CLIP (PAR-CLIP), uses photo-reactive nucleotides and UV-A light for the cross-linking reaction, which increases the incidence of point mutations at the cross-link sites [4]. However, application of PAR-CLIP requires pre-incubation of cells with photoreactive ribonucleoside analogs, and therefore cannot be performed with untreated cells and tissues. The efficiency of nucleoside uptake, and the potential toxicity of these nucleosides [8], might vary between cell lines and tissues. Methods that identify cross-link sites without the need of photo-reactive nucleosides are therefore required.

As originally described by Granneman and colleagues [9], cross-link sites induced by UV-C light are associated with point mutations and deletions in CLIP cDNAs, which was supported by Kishore and colleagues [10]. However, a study by Zhang and Darnell [11] compared the frequency and distribution of deletions and point mutations in CLIP and mRNA-Seq cDNAs, and found that CLIP cDNA deletions were a more reliable signature of cross-link sites compared to point mutations. The cDNA deletions in HITS-CLIP data were then used to identify cross-link sites of Neuro-oncological ventral antigen 1 and 2 (Nova1 and Nova2, which will be together referred to as Nova) and Argonaute (Ago) proteins in a genome-wide manner. Recently, individual-nucleotide resolution CLIP (iCLIP) was developed to identify cross-link sites independently of cDNA mutations [5].

Our first goal was to determine the proportion of truncated cDNAs in the iCLIP cDNA libraries. CLIP and PAR-CLIP protocols identify only the cDNAs that have read through the cross-link site. However, the peptide or amino acid left on the RNA after treatment with proteinase K can obstruct the reverse transcriptase, and therefore primer extension studies showed that a significant proportion of cDNAs truncate at the cross-link sites [12]. iCLIP employs a different cDNA cloning protocol from CLIP and PAR-CLIP, which enables identification of the cDNAs that truncate at the cross-link sites [5]. The position of cDNA truncation therefore enables iCLIP to identify the cross-link sites. The ability of iCLIP to provide nucleotide-resolution information about the cross-link sites was initially demonstrated by determining the positions within uridine tracts that cross-link to heterogeneous nuclear ribonucleoproteins C1/C2 (hnRNP C), and the positions downstream of 5' splice sites that cross-link to cytotoxic granule-associated RNA binding proteins (TIA1 and TIAL1) [5,7]. However, these studies did not evaluate the proportion of cDNAs that truncate at the cross-link sites, as compared to the cDNAs that read through the cross-link sites. If the read-through cDNAs dominated the iCLIP libraries, they could impair the ability of iCLIP to identify the cross-link sites with nucleotide resolution.

Our second goal was to compare the cross-link sites identified by CLIP and iCLIP. Due to the well-characterized sequence preference of Nova proteins and the available CLIP data, we performed iCLIP with Nova proteins in order to compare the two methods. Nova proteins, encoded by *Nova1 *and *Nova2 *genes, contain three KH RNA-binding domains. The sequence specificity of Nova proteins has been extensively characterized using *in vitro *selection and RNA binding, X-ray crystallography, mutagenesis, and computational studies of Nova-dependent splicing enhancer or silencer elements [13-18]. These studies have shown that the KH domains recognize the YCAY motif (Y stands for pyrimidine), such that the affinity of full-length Nova proteins to RNA increases with the number of proximal YCAY tetramers, and a minimum of three to five proximal YCAY tetramers was required for functional binding [13,17]. Analysis of cDNA deletions in Nova CLIP demonstrated that they were located at YCAY motifs, which confirmed that cDNA deletions can identify protein-RNA cross-link sites [11].

Our third goal was to determine the sequence biases of UV-C-induced cross-linking. This question could not be addressed by the past CLIP and iCLIP studies, because all of these studies have used UV-induced cross-linking to identify protein-RNA interactions. We therefore used a method where we induced covalent protein-RNA cross-linking *in vivo *without employing UV-C irradiation. This was achieved by employing the NOP2/Sun domain family, member 2 protein (NSUN2), an RNA methyltransferase that catalyzes the methylation of cytosine to 5-methylcytosine [19-21]. During the catalytic process, cysteine 321 of NSUN2 forms a covalent link with the cytosine residue in the RNA substrate. Cysteine 271 is then required to catalyze release of the methylated RNA from NSUN2. When the cysteine 271 residue is mutated to alanine, release of substrate no longer occurs, and an irreversible covalent bond forms between NSUN2 and RNA [22]. We performed iCLIP with the mutant human NSUN2 (C271A), which allowed us to evaluate the sequence biases introduced by the UV-C induced cross-linking. This demonstrated that both CLIP and iCLIP are subject to a modest uridine preference caused by UV-C cross-linking. In addition, our analyses also demonstrated that CLIP cDNA deletions primarily occur at TTT motifs, and showed that iCLIP cDNA truncation sites analysis is better suited for the study of binding sites located within repetitive motifs.

## Results

### The vast majority of iCLIP cDNAs truncate at the cross-link sites

**Figure 1:**
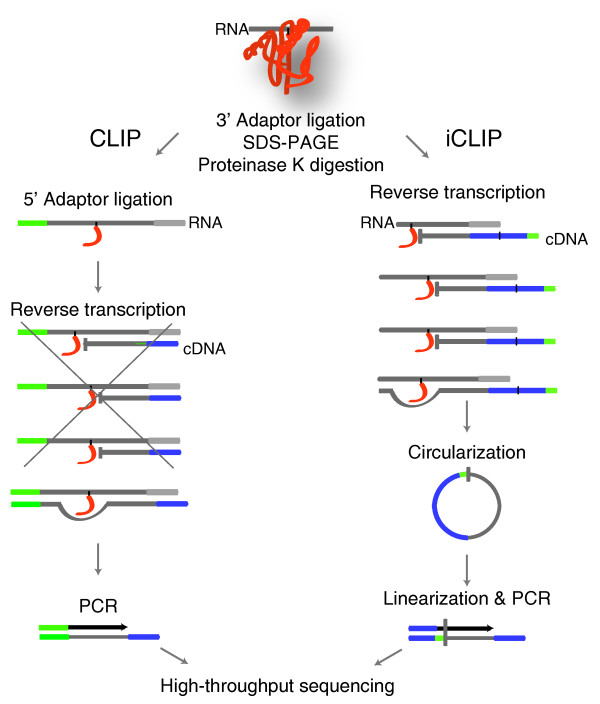
**Schematic summary of CLIP and iCLIP methods**. After UV cross-linking between protein and RNA, both methods purify the protein of interest, ligate the 3' adaptor, purify the protein-RNA complex, and digest the protein by proteinase K. In CLIP, a 5' adapter is ligated to the RNA before reverse transcription. Therefore, CLIP can only amplify cDNAs that read through the cross-link site. However, since the cross-linked nucleotides are covalently bound to the amino acid residue, a proportion of cDNAs truncate at the cross-link site. In iCLIP, truncated cDNAs are captured by circularization and subsequent linearization.

CLIP and iCLIP both employ UV-C irradiation and immunoprecipitation to isolate RNAs cross-linked to a specific protein (Figure 1). Both methods ligate an adapter to the 3' ends of the co-purified RNA fragments. However, the two methods differ in the subsequent steps used to prepare the cDNA library. CLIP ligates an RNA adapter to the 5' ends of the RNA that is later the site for PCR priming. In order to form a molecule competent for PCR amplification, the reverse transcriptase must read through the cross-link site to reach the RNA adapter. However, iCLIP does not ligate an adapter to the 5' ends of RNA, but instead introduces the adapter via an overhang in the primer used for reverse transcription. The adapter is added to the opposite end of cDNAs via circularization, followed by a restriction enzyme cleavage to linearize the cDNAs. This allows amplification of both the truncated and read-through cDNAs. In order to avoid PCR artifacts when quantifying the cDNAs that truncate at the same position, iCLIP also introduced a random barcode into the cDNA adapter [5].

Past studies showed that CLIP cDNAs that read through the cross-link sites had higher proportions of deletions compared to mRNA-Seq cDNAs. Nova CLIP experiments contain a defined proportion of cDNAs with deletions [11]. Therefore, we directly compared the incidence of cDNAs containing deletions in CLIP and iCLIP data to estimate the proportion of truncated cDNAs in iCLIP cDNA libraries. We performed iCLIP for Nova proteins in postnatal mouse brain, using the same antibody and purification protocol as was used in the past studies [11] (Figure s1 in Additional file 1). To avoid the effects of variable sequence read lengths, we evaluated deletions only in the first 25 nucleotides of sequence reads. We used mRNA-Seq to determine the background occurrence of cDNAs containing deletions on our sequencing platform. The deletion ratio in mRNA-Seq was 0.4%, and was therefore compatible with the past study [11]. The proportion of cDNAs containing deletions in Nova CLIP cDNA libraries was 11%, whereas in iCLIP it was only 2.3%, with little variation between experiments (Table 1; Figures s2 and s3 in Additional file 1). Assuming that deletions occur with the same frequency in read-through cDNAs from the CLIP and iCLIP protocols, we estimated that the proportion of read-through cDNAs in Nova iCLIP is approximately 18%, with the remaining 82% representing truncated cDNAs. Among the cDNAs without deletions, which we used to define the cDNA truncation sites, the estimated proportion of truncated cDNAs in Nova iCLIP is 85% (see Materials and methods).

**Table 1:** Deletions in CLIP, iCLIP and mRNA-seq cDNAs

Experiments	Unique cDNAs	Unique cDNAs with deletions in sequence reads	Unique cDNAs with deletions (1 to 25 nucleotides)	Proportion^a^
Nova CLIP	3,852,778	482,871	421,417	11%
Ago mRNA CLIP	1,105,217	79,126	61,211	5.5%
mRNA-Seq	4,857,809	60,110	18,936	0.4%
Nova iCLIP	166,330	6,174	3,749	2.3%
hnRNP C iCLIP	698,046	17,412	8,923	1.3%
TIA1 iCLIP	991,158	6,945	6,261	0.6%
TIAL1 iCLIP	2,786,090	12,011	10,963	0.4%
TDP-43 iCLIP	3,506,515	97,708	48,138	1.4%

^a^Proportion was defined as the percentage of (Unique cDNAs with deletions (1 to 25 nucleotides)/Unique cDNAs).

To analyze if the proportion of truncated cDNAs in iCLIP depends on the protein being studied, we evaluated iCLIP data from past studies of hnRNP C, TIA1, TIAL1 and TAR DNA binding protein (TDP-43; also known as TARDBP) [7,23]. Strikingly, the proportion of cDNAs containing deletions in TIA1, TIAL1 and TDP-43 iCLIP was close to that of mRNA-Seq, indicating that over 95% of cDNAs in these iCLIP experiments truncated at cross-link sites (Table 1; Figures s2 and s3 in Additional file 1). To further consolidate this finding, we evaluated cross-linking of TIA1 and TIAL1 at positions +6 to +30 downstream of exon-intron junctions, which were shown by an independent study to be important for TIA-dependent splicing regulation [24,25]. cDNA truncations identified this region 291 and 457 times more frequently compared to cDNA deletions in TIA1 and TIAL1 iCLIP, respectively (Figure s4 in Additional file 1). This demonstrates the improved capacity of iCLIP cDNA truncations, compared to cDNA deletions, in identifying the TIA binding sites. Taken together, our results indicate that the vast majority of cDNAs in iCLIP experiments are truncated at the protein-RNA cross-link sites.

### Analysis of sequence biases at the cross-link sites identified by CLIP or iCLIP

**Figure 2:**
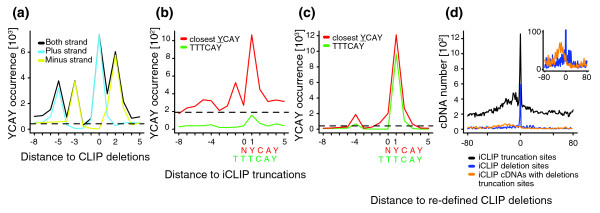
**iCLIP cDNA truncations identify RNA cross-link sites of Nova proteins with nucleotide resolution**. **(a) **The occurrence of YCAY motifs around the CLIP cDNA deletions (FDR < 0.001). The black line shows the starting position of YCAY motifs on all cDNAs, whereas the light blue and yellow lines show the starting position on the plus or minus strand of the genome. The dashed line shows the background occurrence of YCAY motifs. **(b) **The occurrence of YCAY motifs starting closest to the iCLIP cDNA truncations (FDR < 0.05). The red line shows the starting position of YCAY motifs, and the green line shows the starting position of TTTCAY motifs. **(c) **Similar to (b), but the occurrence of YCAY motifs starting closest to redefined CLIP cDNA deletions, where the position of deletions mapping within TTT motifs is assigned to the middle of TTT. The red line shows the starting position of YCAY motifs, and the green line shows the starting position of TTTCAY motifs. **(d) **Occurrence of iCLIP cDNA truncations (black), deletions (blue) or truncations of cDNA with deletions (orange) around the re-defined CLIP deletion sites. The number of cDNAs was determined by considering the random barcode. iCLIP cDNA deletion sites were re-defined as described in Materials and methods.

As described earlier, the specificity of Nova proteins for YCAY clusters has been extensively studied. Therefore, we compared the ability of CLIP and iCLIP to identify such clusters. First, we compared the position of Nova CLIP cDNA deletion sites as determined by Zhang and Darnell [11] with the Nova iCLIP cDNA truncation sites as determined by the experiments conducted for this study. cDNA truncation sites were defined by the nucleotide following the 3' end of cDNAs (that is, the nucleotide preceding the sequence reads), which was assigned the position 0 in the present analyses of iCLIP data. For identification of significant sites, we employed the same methods as used in the original studies (false discovery rate (FDR) < 0.001 for CLIP cDNA deletions [11] and FDR < 0.05 for iCLIP cDNA truncations [7]). We then plotted the occurrence of YCAY motifs relative to the positions of cross-link sites identified by the two methods. Surprisingly, this indicated different positioning of YCAY motifs around cross-link sites defined by CLIP and iCLIP (Figure 2a; Figure s5A in Additional file 1). As reported previously, the occurrence of YCAY motifs peaked at positions -5, -3, 0 and +2 relative to the CLIP cDNA deletion sites (Figure 2a) [11]. In contrast, YCAY motifs peaked at position +1 relative to the iCLIP cDNA truncation sites (Figure s5A in Additional file 1). To understand this discrepancy, we evaluated the CLIP cDNA deletion sites in more detail. We found that the distance between YCAY motifs and the deletions in CLIP cDNAs that mapped to the two strands of the genome were shifted by two nucleotides relative to each other (Figure 2a). One possible cause of this shift could be the strong preference of cDNA deletions for TTT motifs (Figure s6A-C in Additional file 1). Since cDNA deletions cannot be positioned within such mononucleotide repeats, Novoalign, a program that was used for the mapping of deletion sites, automatically assigns the position of deletion to the 3' end of TTT motifs in the transcripts on the plus strand of the genome, and to the 5' end of TTT motifs in the transcripts on the minus strand. We therefore re-defined the deletion sites within TTT motifs to the center of this motif, which completely corrected the mismatch between the two strands, and showed that the YCAY motif positioning was different from that observed in the original study [11] (Figure s5B in Additional file 1). We used these re-defined positions for the remaining analyses of Nova cDNA deletions in this study.

The re-defined positions of cDNA deletions showed that YCAY motifs were enriched only at positions -4 and +1 relative to the deletion sites (Figure s5B in Additional file 1). Notably, the vast majority of these cDNA deletions were located within TTT motifs (Figure s6 and Additional file 1), and TTT enrichment was present also at Ago CLIP cDNA deletion sites (Figure s7 in Additional file 1). Furthermore, TTT enrichment was present at Nova CLIP cDNA deletion sites even if we did not use an FDR threshold to define the significant CLIP cDNA deletion sites (Figure s6D, E in Additional file 1). The TTTCAY motif represented 80% of the Nova CLIP cDNA deletions that mapped to the nucleotide preceding the YCAY motif (+1 position; Figure 2c), and YCATTT represented 90% of the cases where cDNA deletions mapped to the nucleotide following the YCAY motif (-4 position; Figure s5D in Additional file 1). Furthermore, the YCATTTCAY motif represented 56% of the cases where CLIP cDNA deletions mapped to the -4 position of YCAY (Figure s5B in Additional file 1), indicating that the -4 peak was largely a result of the TTT enrichment at CLIP cDNA deletions. Therefore, we evaluated only the YCAY motif starting closest to each cross-link site, which showed that CLIP cDNA deletions and iCLIP cDNA truncations both identified the nucleotide preceding the YCAY motifs (+1 site) as the primary Nova cross-link site (Figure 2b, c). Importantly, TTTCAY represented only 15% of the cases where Nova iCLIP cDNA truncations mapped to the nucleotide preceding the YCAY motif (+1 position; Figure 2b) and 22% of the cases where iCLIP cDNA truncation mapped to the nucleotide following the YCAY motif (-4 position; Figure s5C in Additional file 1). Nova and Ago proteins do not have a known binding preference for the U tracts. Therefore, the enrichment of the TTT motif is most likely associated with the deletion sites in read-through cDNAs. The analysis of cDNA truncations in iCLIP therefore provides an advantage by identifying cross-link sites lacking the TTT motif.

### iCLIP cDNA truncations identify the positions of CLIP cDNA deletions

To further examine the overlap between cross-link sites identified by CLIP and iCLIP, we directly compared the positions of the re-defined cDNA deletions in CLIP (FDR < 0.001) and cDNA truncations in iCLIP (no FDR threshold). iCLIP cDNA truncation sites were significantly enriched at the CLIP deletion sites, confirming that iCLIP cDNAs represent truncations at the cross-link sites (Figure 2d; Figure s8A, B in Additional file 1). In contrast, the 3' ends of CLIP cDNAs that lack deletions did not overlap with the CLIP deletion sites, confirming that the overlap is specific to iCLIP libraries (Figure s8C, D in Additional file 1). Similarly, the 3' ends of iCLIP cDNAs containing deletions did not overlap with the CLIP cDNA deletion sites (Figure 2d; Figure s8E in Additional file 1). Instead, the 3' ends of iCLIP cDNAs containing deletions had a similar pattern to the 3' ends of CLIP cDNAs, and iCLIP cDNA deletion sites were significantly enriched at CLIP cDNA deletion sites, indicating that most iCLIP cDNAs containing deletions represent read-through sequences (Figure 2d; Figure s8 in Additional file 1). In conclusion, we find that iCLIP cDNAs lacking deletions truncate at positions overlapping with deletions in CLIP or iCLIP cDNAs, confirming that they can identify the position of cross-link sites.

### UV-C-induced cross-linking preferentially occurs at uridines

**Figure 3:**
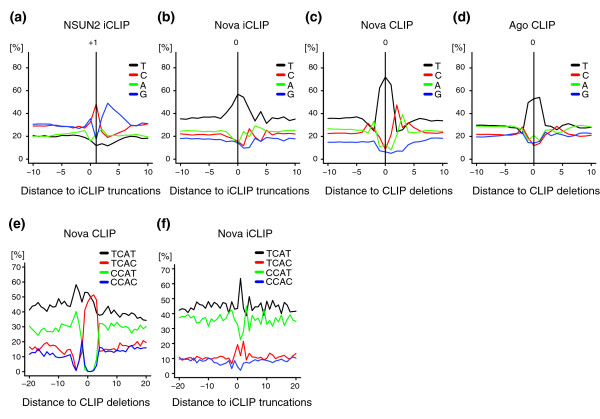
**Enrichment of T nucleotide at cross-link sites induced by UV-C**. **(a) **The nucleotide compositions around NSUN2 iCLIP cDNA truncations on the protein-coding genes (including introns). **(b) **Similar to (a), but iCLIP with Nova protein. **(c) **Similar to (a), but around Nova CLIP cDNA deletions. The deletion sites were re-defined as described in the main text. **(d) **Similar to (c), but around Ago CLIP cDNA deletions. **(e) **The proportion of the four types of YCAY motif around Nova CLIP cDNAs deletions. **(f) **Similar to (c), but around Nova iCLIP cDNA truncations.

To conduct a comprehensive analysis of sequence biases that might be associated with UV-C-induced cross-linking, we performed iCLIP with the mutant NSUN2 (C271A), which forms an irreversible covalent bond with cytosine without the need of UV-C-induced cross-linking [22]. Analysis of the NSUN2 iCLIP cDNA libraries showed strong cytosine enrichment at position +1 (Figure 3a), which corresponds to the 3' end of cDNAs (that is, the first nucleotide of the sequence reads). On the other hand, analysis of iCLIP data of five other proteins showed thymidine enrichment at position 0, which contrasted a depletion of thymidines at the same position in NSUN2 iCLIP (Figure 3a, b; Figure s9 in Additional file 1). These results demonstrate that the nucleotide enrichment at cross-link sites reflects the cross-linking protocol used; the spontaneous cross-linking of NSUN2 predominantly occurs at cytosines, whereas UV-C cross-linking predominantly occurs at uridines. However, binding preferences also contribute to the nucleotide enrichments, as seen by a stronger thymidine enrichment at cross-link sites of proteins that directly bind uridine tracts (hnRNP C, TIA1 and TIAL1) compared to Nova and TDP-43 proteins, which are not thought to bind uridine tracts (Figure 3b; Figure s9 in Additional file 1). It is clear, however, that the uridine bias resulting from UV-C cross-linking is modest compared to the enrichment of cDNA deletions at TTT motifs (Figure 3b-d; Figure s9 in Additional file 1).

### The use of cross-link sites to study RNA binding specificity

To evaluate how the sequence biases at cross-link sites influence the study of RNA binding specificity of Nova, we assessed the nucleotide composition of the two variant pyrimidine positions of YCAY motifs at Nova cross-link sites identified by the CLIP cDNA deletions or iCLIP cDNA truncations (Figure 3e, f). We found that the relative proportions of TCAC increased at cross-link sites of both methods, with a corresponding decrease in the CCAT motif (Figure 3e, f). To quantify this change, we compared the ratio of YCAY motifs starting at positions 0 to +2 to those starting at positions -20 to +20. At the CLIP cDNA deletion sites, CCAT decreased from 21% to 0.3%, whereas at iCLIP cDNA truncation sites the decrease was from 36% to 26% with a corresponding increase in TCAC (Figure s10A-D in Additional file 1). This indicates that the analysis of sequence motifs at cross-link sites identified by CLIP cDNA deletions has stronger sequence preferences compared to cross-link sites identified by iCLIP cDNA truncations.

### iCLIP allows quantitative analysis of protein occupancy on its RNA-binding sites

**Figure 4:**
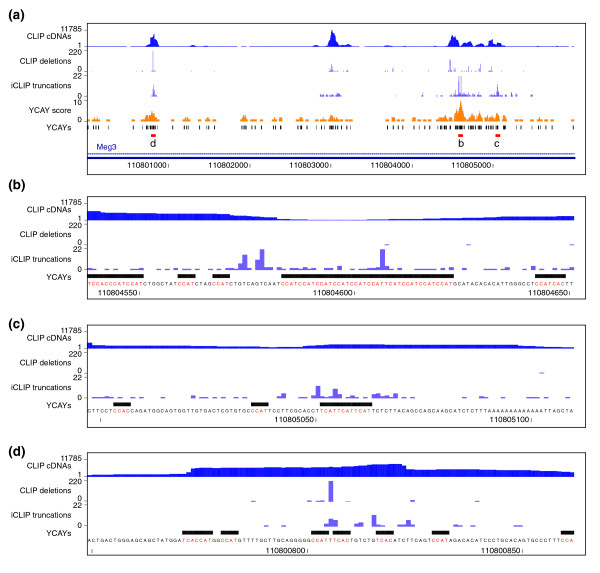
**Comparison of CLIP and iCLIP analysis of Nova binding to its primary RNA target, the *Meg3 *non-coding RNA**. **(a) **Overview of a region in *Meg3 *non-coding RNA (chr12: 110800000-110806000). In each panel, the CLIP cDNAs track shows the cluster of CLIP cDNAs without deletions. The CLIP deletions track shows low FDR deletion sites (FDR < 0.001, the positions were re-defined), with peak height corresponding to the number of sequences containing deletions at the sites. The iCLIP truncations track shows the position of iCLIP cDNA truncations (FDR < 0.05), with peak height corresponding to the cDNA counts. The YCAY score track shows the YCAY score at each position, while the YCAYs track shows the position of YCAY motifs. **(b) **Region with the highest iCLIP cDNA count. This region also has the highest YCAY score. **(c) **Region with the second highest iCLIP cDNA count. **(d) **Region with the highest number of CLIP cDNA deletions. Same scale is used in all panels in order to allow comparisons of the evaluated binding sites.

To compare the ability of CLIP and iCLIP to monitor the relative occupancy of an RBP on different RNA-binding sites, we evaluated cross-link sites determined by the two methods in the *Meg3 *gene (also known as *Gtl2*). *Meg3 *is a maternally expressed non-coding RNA and thought to be involved in mouse embryonic development [26]. Zhang *et al. *[27] showed that the human ortholog, *MEG3*, has 12 alternative splicing variants. *Meg3 *contains approximately 3% of all Nova CLIP cDNAs [1], and is therefore the RNA with strongest cross-linking to Nova in mouse brain. The overall high coverage in the *Meg3 *RNA allows analysis of cDNA counts at individual binding sites. As described previously, Nova functional binding sites are composed of multiple closely spaced YCAY motifs, also referred to as YCAY clusters, such that the Nova affinity for RNA correlates with the number of proximal YCAY motifs [13,17]. Therefore, to identify candidate high-affinity Nova binding sites, we calculated the YCAY cluster score by counting the number of YCAY motifs in 41 nucleotide sliding windows (Figure 4a). We then compared the YCAY cluster score with cDNA counts at cross-link sites identified by CLIP cDNA deletions or iCLIP cDNA truncations. Interestingly, whereas YCAY cluster scores correlated poorly with CLIP cDNA deletion counts (Spearman's rho = 0.25, *P*-value = 0.16), correlation with iCLIP cDNA counts was highly significant (Spearman's rho = 0.53, *P*-value = 0.0013) (Figure 4a-c). Accordingly, the regions with the highest YCAY cluster score contained the highest iCLIP cDNA counts. On the other hand, the strongest site defined by CLIP cDNA deletions resided within a TTT motif positioned between two YCAYs, even though it was not part of a highly scoring YCAY cluster (denoted 'd' in Figure 4a, d).

**Figure 5:**
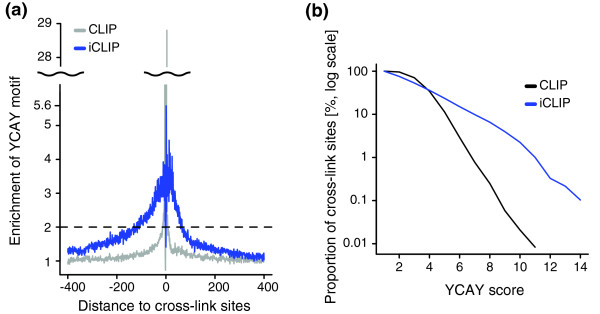
**Comparison of the ability of CLIP and iCLIP to identify clustered YCAY motifs**. **(a) **The enrichment of the YCAY motif around cross-link sites starting at each position relative to cross-link sites identified by re-defined CLIP cDNA deletions and iCLIP cDNA truncations. Both peaked at position +1, where the enrichment factor was 29 for CLIP cDNA deletions and 5.6 for iCLIP cDNA truncations. The dashed line shows two-fold enrichment of the YCAY motif compared to background. **(b) **The proportion of cross-link sites with YCAY score greater than or equal to the value shown on the x-axis.

The observations above strongly suggested that the quantitative information in iCLIP corresponds to the affinity of Nova for its binding sites. In order to test whether the greater ability of iCLIP to identify YCAY clusters is evident also in other RNA targets, we evaluated the enrichment of YCAY motifs in the region surrounding the cross-link sites. A greater than two-fold enrichment of YCAY motifs was restricted to the area from -12 to +8 nucleotides surrounding the CLIP cDNA deletions, and from -118 to +65 nucleotides surrounding the iCLIP cDNA truncations (Figure 5a). Interestingly, the only YCAY tetramers enriched in the region surrounding the cross-link sites were TCAT and CCAT (Figure s10E-G in Additional file 1). Motif enrichment in the region surrounding the cross-link sites identified by iCLIP indicates the presence of highly clustered YCAY motifs. Next, we evaluated the ability of CLIP and iCLIP to detect YCAY clusters. We evaluated cross-link sites present in YCAY clusters of different lengths, which confirmed that iCLIP was better capable of identifying cross-link sites with higher YCAY scores compared to CLIP (Figure 5b). In conclusion, our analysis indicates that the quantitative information of iCLIP corresponds well to the YCAY scores, and is better capable of identifying the highly clustered binding motifs.

## Discussion

In this manuscript, we benchmarked CLIP and iCLIP, the two most frequently used methods for transcriptome-wide study of protein-RNA interactions in untreated cells and tissues. We showed that similarly to CLIP, iCLIP libraries contain a small proportion of cDNAs with deletions. Therefore iCLIP can identify cross-link sites by two independent approaches: cDNA deletions or cDNA truncations. Even though the proportion of iCLIP cDNA with deletions is very low, the overlap of deletions with the cross-link sites identified by cDNA truncations can serve to validate the nucleotide resolution of iCLIP data. The low proportion of cDNAs with deletions indicates that 82% of Nova iCLIP cDNAs were truncated at cross-link sites, and this proportion is even greater in iCLIP data of other proteins. The variable proportions of truncated cDNAs in iCLIP of different RBPs might reflect the effects of different peptides that remain bound to the RNA after proteinase K digestion. Since iCLIP can produce both truncated and read-through cDNAs, it can robustly identify RNA-binding sites even in cases where the read-through cDNAs are rarely produced (such as in the TIA proteins), and is therefore capable of studying a larger repertoire of RBPs. Furthermore, by using the mutant NSUN2 protein, we demonstrated that iCLIP can identify cross-link sites induced either by UV-C-induced cross-linking or other covalent cross-linking protocols.

We found that the TTT motif was the primary motif at the cross-link sites identified by Nova and Ago CLIP cDNA deletions. Since these studies did not identify recognition of uridine-rich sequences by Nova or Ago proteins, the potential functional relevance of the TTT motif remains to be established. Importantly, we found that the TTT motif is not enriched in Nova CLIP cDNAs without deletions, which constitute the large majority of CLIP cDNAs (Figure s11 in Additional file 1), indicating that the enrichment of TTT might be a bias introduced by the cDNA deletion analysis. As has been shown in past studies of the slippage-mediated mutations by HIV reverse transcriptase, one-base deletions are most common at homonucleotide runs [28]. Therefore, the increased incidence of cDNA deletions at homonucleotide runs, together with the UV-C cross-linking bias for uridines, might be responsible for the enrichment of TTT motif at the cross-link sites identified by cDNA deletions in Nova and Ago CLIP. It remains to be seen if the TTT motif is the primary site for deletions only in Nova and Ago CLIP cDNAs, or also in CLIP of other RBPs.

It is also important to be aware that cDNA mutations in CLIP and PAR-CLIP may represent genomic variation rather than cross-link induced mutations. For instance, we found that most deletions in TDP-43 iCLIP cDNAs constituted consecutive dinucleotide deletions in TG repeats (Figure s12 in Additional file 1), unlike the deletions in Nova CLIP cDNAs where consecutive dinucleotide deletions constituted only 21% of all deletions [11]. Such dinucleotide variation is common in the human genome because TG repeats correspond to the hyper-variable CA microsatellite. Thus, it is likely that most deletions identified in TDP-43 iCLIP cDNAs are a result of genomic variation, rather than cross-link-induced mutations. Methods that aim to identify cross-link sites by analysis of mutations in cDNAs are therefore prone to identifying genomic variation instead of cross-link sites. Analysis of cDNA truncations in iCLIP is therefore useful to identify cross-link sites independent of the genomic variation.

To evaluate the nucleotide preferences of UV-C-induced cross-linking, we compared it with the spontaneous covalent cross-linking of NSUN2. We observed a consistent T enrichment at position 0 of all iCLIP studies where cross-linking was induced with UV-C - since this nucleotide is not part of cDNAs (but is upstream of cDNAs), the T enrichment could only result from steps up to reverse transcription that are common between CLIP and iCLIP. Moreover, NSUN2 had no T enrichment, but instead had C enrichment at position +1. This indicates that UV-C-induced cross-linking has a uridine bias. As data of additional RBPs become available, other nucleotide biases might be identified. Our results also indicate that cDNAs can truncate either one nucleotide before the cross-link sites, as appears most common in the case of UV-C-induced cross-linking, or directly at cross-link sites, as is most common in the case of NSUN2.

Since the methylation by NSUN2 is a transitory enzymatic reaction, we could not cross-link NSUN2 by UV-C light in order to directly compare the cross-link sites of the different methods. Instead, we compared cross-link sites identified by cDNA deletions in Nova CLIP and cDNA truncations in Nova iCLIP. The sequence specificity of Nova proteins has been extensively characterized by previous evolutionary conservation [29] and affinity measurements [13-18]. Both our and previous studies [11] showed that both TCAT and CCAT are highly enriched in the region surrounding the cross-link sites. However, there is a large change in the proportion of TCAT and CCAT enrichment at deletion sites of CLIP cDNAs, which is consistent with our finding that deletions primarily occurred at the TTT motif. In contrast, there is a small change in TCAT and CCAT at iCLIP cDNA truncation sites, which likely reflects the uridine preference of UV-C cross-linking. This indicates that the enriched sequence motifs at cross-link sites identified by CLIP are more strongly affected by the sequence preferences of cDNA deletions compared to iCLIP cDNA truncation sites.

It is clear that the motifs enriched directly at cross-link sites need to be interpreted with caution because of the potential effects of nucleotide preferences of UV cross-linking. However, we demonstrate that enrichment of the sequence motifs recognized by each RBP is not restricted to the cross-link sites. This is particularly evident by the enrichment of TCAT and CCAT in Nova iCLIP, and TG repeats in TDP-43 iCLIP, which is present even at a distance of over 20 nucleotides away from the cross-link sites (Figures s10E-G and s11 in Additional file 1). This pattern of enrichment most likely reflects the high-affinity binding sites of RBPs, which are often composed of clusters of short motifs [23,30]. Analysis of such clustered motifs that are enriched not only directly at the cross-link sites but also in the vicinity of cross-link sites could avoid the sequence biases of deletion site analysis or UV-C-induced cross-linking.

Past studies summarized the CLIP data at multiple binding sites across the genome to show that they provide quantitative information [10]. However, it was not clear if occupancy of individual binding sites within an individual RNA could be quantitatively compared. We analyzed the primary Nova RNA target *Meg3*, which showed that iCLIP cDNA counts correlate well with the YCAY cluster score. The use of random barcode for cDNA quantification [5] is one reason for the increased quantitative nature of iCLIP. Moreover, genome-wide analysis showed that iCLIP identifies a larger number of clustered YCAY motifs. This difference may be explained by the lack of TTT preference in iCLIP, or the increased mappability of iCLIP cDNAs, since the truncated cDNA are less likely to fully overlap with the repetitive motif clusters. Although we showed that iCLIP truncation analysis allows the comparison of binding sites within a single transcript, care needs to be taken in comparisons of binding sites on different transcripts, and between exons and introns of a transcript, because these can vary dramatically in their abundance. The accessibility of an RBP to different transcripts also depends on its localization within the cell. The normalization approaches to take these variations into account have been recently reviewed [31]. Our study indicates that UV-C cross-linking is associated with a mild uridine bias, which can be avoided by analysis of the motifs enriched in the vicinity of cross-link sites.

## Conclusions

Our analysis showed that over 80% of cDNAs were truncated at cross-link sites. We showed that cDNA truncations in iCLIP can identify the same cross-link sites as CLIP cDNA deletions. Moreover, since only iCLIP can recover truncated cDNAs, iCLIP identifies cross-link sites more comprehensively. We observed a strong enrichment of the TTT motif at CLIP cDNA deletion sites, but only a mild T enrichment at iCLIP cDNA truncation sites. The T enrichment most likely results from uridine preference of UV-C-induced cross-linking, because it is absent when we perform UV-independent cross-linking of a mutant RNA methylase. The TTT enrichment, however, most likely results from analysis of cDNA deletions, because it is absent when analyzing CLIP cDNAs without deletions. Finally, we demonstrated that iCLIP is better capable of identifying long YCAY clusters as the primary Nova binding sites.

## Materials and methods

### CLIP, mRNA-Seq and iCLIP data sets and experiments

Nova and Ago CLIP data sets [2,32,33] and the significant cDNA deletion sites were described by Zhang and Darnell [11]. The cDNA library of mRNA-Seq for HeLa cell transcripts was prepared using an Illumina TruSeq kit. Nova iCLIP was performed by following the standard iCLIP protocol for brain tissue [5,23]. We used postnatal mouse brain tissue and immunoprecipitated Nova protein using an anti-Nova antibody [1]. hnRNP C, TIA1, TIAL1 and TDP-43 iCLIP data sets were available from past studies [5,7,23]. For NSUN2 iCLIP, we followed the standard iCLIP protocol with the following modifications: we transfected COS7 cells with the C271A mutant NSUN2, and did not subject the cells to UV-C irradiation. We immunoprecipitated the mutant NSUN2 using an antibody against the myc epitope tag (9E10; Sigma-Aldrich, St. Louis, MO, USA). High-throughput sequencing for the experiments conducted in this study was performed using the Illumina Genome Analyzer IIx.

### Mapping and annotation of sequencing data

We used the mm9/NCBI37, hg19/GRCh37 and MGSC Merged 1.0/rheMac2 genome assemblies and Ensembl 59 (for mouse and human) and Ensembl63 (for rhesus macaque) gene annotation. Before mapping we removed random barcode and adaptor sequences from iCLIP cDNA sequences, as described previously [5]. We performed iterative mapping of cDNAs without deletions, followed by mapping of remaining cDNAs containing deletions. In the first round, we mapped the cDNAs to the genome with Bowtie 0.12.7 [34], which does not allow deletions, using the following parameters: -v 2 -m 1 -a --best --strata. The nucleotide preceding the iCLIP cDNAs mapped by Bowtie was used to define the cross-link sites identified by truncated cDNAs. In the second round, we mapped the remaining cDNAs to the genome using Novoalign [35], which can map cDNAs containing deletions, using the following parameter: -e 0. The deleted nucleotide in CLIP and iCLIP cDNAs mapped by Novoalign was used to define the cross-link sites identified by read-through cDNAs. If a cDNA had more than one deletion, we selected the one closest to the beginning of the read. When multiple cDNAs with the same random barcode mapped to the same starting position in the genome, but contained deletions at different sites, we selected the deletions with most frequent occurrence. If two deletions had the same frequency of occurrence, we selected the one closest to the beginning of the sequence read for the cDNAs. If the cDNA did not contain random barcode (CLIP and mRNA-Seq), we followed a procedure where we allocated the same random barcode to all cDNAs. The method for the random barcode evaluation, annotation of genomic segments and identification of significantly clustered cDNA truncation sites was described earlier [5,7], except that the Ensembl 59 gene annotation was used. For analyses of CLIP, mRNA-Seq and iCLIP data, we only used cDNA libraries that contained more than 10,000 uniquely mapped reads.

### Calculating the number of total cDNAs and cDNAs with deletions

Since CLIP and mRNA-Seq cDNA lacked random barcodes, for the comparison of the number of total cDNAs or cDNAs with deletions in CLIP, mRNA-Seq and iCLIP cDNA library (Table 1), we performed the following procedure to cancel random barcode evaluation of iCLIP libraries. For total cDNA number calculations, we joined all sequence reads starting at the same position of the genome into a single read. For cDNAs with deletions, we selected unique cDNAs with deletions as described above. If there was more than one cDNA with deletions, where the sequence reads started the same position of the genome, we joined them and defined the deletion sites as the one closest to the beginning of the reads. This analysis and all following analyses were done with custom Python and R scripts and the iCount server [36].

### Calculating the proportion of read-through cDNAs in Nova iCLIP cDNA libraries

First, we estimated the proportion of read-through cDNAs in the total iCLIP cDNA library by evaluating the proportion of cDNAs containing deletions. This allows us to evaluate the proportion of cDNAs that were missed in the CLIP protocol due to cDNA truncations. In this we assumed the following: first, Nova CLIP cDNA libraries contain only read-through cDNAs, whereas Nova iCLIP cDNA libraries contain read-through and truncated cDNAs; and second, due to the identical protocol for reverse transcription and sequencing, the rate of deletions and their distribution in read-through cDNAs was the same in Nova CLIP and iCLIP cDNA libraries.

Furthermore, while both CLIP and iCLIP aim to prepare libraries with average cDNA lengths of 50 nucleotides, different experiments had some variation in sequence lengths. To avoid this variation when comparing cDNA libraries of CLIP, mRNAseq and iCLIP, we only evaluated deletions in the first 25 nucleotides from the 5' end of cDNAs.

Thus, we estimated *f*, the proportion of read-through cDNAs in the total Nova iCLIP library, to be 18% according to the following formula:

$$\begin{array}{c} p\left(iCLIP\right)=f\times p\left(RT\right)+\left(1-f\right)\times p\left(BG\right)\\ \Leftrightarrow f=\frac{p\left(iCLIP\right)-p\left(BG\right)}{p\left(RT\right)-p\left(BG\right)}\approx \text{0}\text{.177} \end{array}$$

To make this estimate we used the following values: *p(iCLIP)*, the proportion of cDNAs with deletions in the first 25 nucleotides for Nova iCLIP data (3,749/166,330, ≈2.3%); *p(RT)*, the proportion of cDNAs with deletions in the first 25 nucleotides for read-through cDNAs from Nova CLIP data (421,417/3,852,778, ≈11%); and *p(BG)*, the proportion of cDNA with deletions in the first 25 nucleotides of mRNA-Seq cDNAs, which we used to estimate the background occurrence of deletions (18,936/4,857,809, ≈0.4%). Thus, we estimated that 82% of cDNAs were lost in CLIP cDNA cloning protocol due to truncations.

Since read-through cDNAs have a high incidence of deletions, the cDNAs with deletions are not informative for analysis of cDNA truncations. Therefore, we only studied cDNAs without deletions in this and in past publications for cross-link site identification by iCLIP. It is important to determine the proportion of truncated cDNAs among the iCLIP cDNAs without deletions; 3.7% (6,174/166,330) of Nova iCLIP cDNAs contained a deletion in the sequence read. These deletions can occur in both read-through cDNAs and truncated cDNAs. We estimate that 86% of these deletions occurred in read-through cDNAs according to the following formula (the proportion is denoted as *k*):

$$k=\frac{f\times p\left(CLIP\right)}{f\times p\left(CLIP\right)+\left(1-f\right)\times p\left(BG\right)}\approx \text{0}\text{.858}$$

The estimated proportion of read-through cDNAs among Nova iCLIP cDNA library without deletions in the sequence reads was 15% according to the following formula:

$$\frac{\left(\text{total cDNA}\right)\;\times f-\left(\text{cDNA}\text{ with deletions}\right)\times k}{\left(\text{total cDNA}\right)-\left(\text{cDNA with deletions}\right)}\approx \text{0}\text{.150}$$

Thus, we estimate that 85% of Nova iCLIP cDNAs, among the cDNAs that lack deletions, were truncated at cross-link sites.

### Re-defining the deletion sites

We searched the sequence from -2 to 0 positions to the deletion sites for the plus strand-mapped cDNAs and from 0 to 2 for the minus strand-mapped cDNAs. If the sequence was TTT, we re-defined the deletion site as the middle of the TTT motif. If the re-defined deletion site overlapped with another existing deletion site, the deletion counts were summed. Nucleotide composition around deletion sites was visualized with WebLogo 3 [37].

### YCAY motif occurrence and enrichment around cross-link sites

The YCAY motif occurrence was calculated around cross-link sites defined by confident CLIP deletion sites by Zhang and Darnell (FDR < 0.001) [11], or by confident iCLIP truncation sites (FDR < 0.05) [7]. The cross-link sites were evaluated on the sense strand of transcribed regions and on both strands of the intergenic regions. The closest YCAY motif was defined by recording the starting position of the YCAY motif with the smallest distance to the cross-link site. If two YCAY motifs had the same distance to cross-link sites, we selected the upstream motif (for example, if the closest YCAY motifs started at positions -5 and +5, we selected only the position -5). To determine the background occurrence of YCAY motifs, we randomly re-positioned the cross-link sites within the same genomic segment (for instance, in the same 3' untranslated region or the same intron, as described before [7]) and calculated YCAY occurrence around these re-positioned sites (in the region -50 to 50 relative to the sites). We performed this randomization 100 times and calculated the average background YCAY motif occurrence. To determine the region of two-fold enrichment in Figure 5a, we averaged the enrichment at -2 to +2 positions around each position to avoid the effects of fluctuations.

### Visualization of cDNAs and cross-link sites on the *Meg3 *RNA

We used the postnatal mouse brain Nova CLIP data set to visualize the Nova CLIP cDNAs without deletions for Figure 4. The cDNAs without deletions were mapped with Bowtie as described above (without a FDR threshold), and converted to eland format. The cDNAs were then clustered with the Findpeaks 3.1.9.2 program [38] using the following argument: -dist_type 0 50 -hist_size 1 -eff_size 1.8655e9. The Nova CLIP cDNA deletion sites (FDR < 0.001) and the counts were described above and re-defined as described above. The Nova iCLIP truncation sites (FDR < 0.05) and the cDNA counts were described above. These data sets on the *Meg3 *gene were visualized with the UCSC genome browser.

### Calculation of the YCAY score

The YCAY score corresponds to the density of YCAY motifs in a 41-nucleotide sliding window. A region comprising 20 nucleotides upstream and downstream around the genomic position of interest was evaluated, and the number of YCAY motifs that were completely contained in the area was used to determine the YCAY score for the position.

### Correlation between YCAY score and CLIP or iCLIP cDNA counts

The region chr12:110796849-110809936 on the mouse genome (mm9) was evaluated to study Nova binding to the *Meg3 *RNA. The correlation between the YCAY score of the YCAY cluster and the highest CLIP or iCLIP cDNA counts in the cluster was calculated.

The YCAY clusters were defined using an approach inspired by the Findpeaks 3.1.9.2 program [38]: 1) calculate the YCAY score for all positions in the region and determine the local maximum; 2) if the minimum score between local maxima was 0, the clusters ended at the position where the score became 0; 3) if the minimum score between the local maxima was not 0, compare the minimum score with 0.9-fold of the smaller of the two local maxima; 4) if the minimum score was smaller, separate the cluster at the middle of the area with the local minimum value; 5) if the minimum score was larger, join the two peaks into the same cluster, and compare its local maximum to the next local maximum, starting from step 2.

The maximum YCAY score in each cluster was defined as the YCAY score of the cluster. We only used the clusters that contained at least one cross-link site to calculate the correlation. We calculated the Spearman's rank correlation coefficient between the YCAY score and cDNA count. The same analysis was done to calculate the correlation with cDNA counts at cross-link sites defined by either CLIP cDNA deletions or iCLIP cDNA truncations.

### Statistical analysis

The *P*-value of the correlation between the YCAY score and cDNA count of iCLIP or CLIP on the region of the *Meg3 *RNA described above was calculated using asymptotic t approximation as two-sided. These value was calculated with cor.test(x, y, alternative = "two.sided", method = "spearman", exact = FALSE) function of R.

### Data access

The Nova and NSUN2 iCLIP data are available from ArrayExpress with accession number E-MTAB-1008 and together with past published iCLIP data also from iCount [36].

## Abbreviations

Ago: Argonaute; CLIP: UV cross-linking and immunoprecipitation; FDR: false discovery rate; HITS-CLIP: high-throughput sequencing of RNA isolated by crosslinking immunoprecipitation; hnRNPC: heterogeneous nuclear ribonucleoproteins C1/C2; iCLIP: individual-nucleotide resolution UV cross-linking and immunoprecipitation; Nova: Neuro-oncological ventral antigen; NSUN2: NOP2/Sun domain family, member 2; PAR-CLIP: Photoactivatable Ribonucleoside-Enhanced CLIP; RBP: RNA-binding protein; TDP-43: TAR DNA binding protein (also known as TARDBP); TIA1: cytotoxic granule-associated RNA binding protein; TIAL1: TIA1-like 1.

## Competing interests

The authors declare that they have no competing interests.

## Authors' contributions

JK and SH performed the experiments, YS and TC analyzed the data, YS and JU wrote the paper, and BZ, MF and JU supervised the study. All authors read and approved the final manuscript for publication.

## Supplementary Material

Additional file 1 **Supplementary Figures s1 to s12**. Click here for file

## References

[B1] Ule J Jensen KB Ruggiu M Mele A Ule A Darnell RB CLIP identifies Nova-regulated RNA networks in the brain. Science 2003 13 1212 1215 10.1126/science.1090095 14615540

[B2] Licatalosi DD Mele A Fak JJ Ule J Kayikci M Chi SW Clark TA Schweitzer AC Blume JE Wang X Darnell JC Darnell RB HITS-CLIP yields genome-wide insights into brain alternative RNA processing. Nature 2008 13 464 469 10.1038/nature07488 18978773 PMC2597294

[B3] Yeo GW Coufal NG Liang TY Peng GE Fu XD Gage FH An RNA code for the FOX2 splicing regulator revealed by mapping RNA-protein interactions in stem cells. Nat Struct Mol Biol 2009 13 130 137 10.1038/nsmb.1545 19136955 PMC2735254

[B4] Hafner M Landthaler M Burger L Khorshid M Hausser J Berninger P Rothballer A Ascano M Jr Jungkamp AC Munschauer M Ulrich A Wardle GS Dewell S Zavolan M Tuschl T Transcriptome-wide identification of RNA-binding protein and microRNA target sites by PAR-CLIP. Cell 2010 13 129 141 10.1016/j.cell.2010.03.009 20371350 PMC2861495

[B5] Konig J Zarnack K Rot G Curk T Kayikci M Zupan B Turner DJ Luscombe NM Ule J iCLIP reveals the function of hnRNP particles in splicing at individual nucleotide resolution. Nat Struct Mol Biol 2010 13 909 915 10.1038/nsmb.1838 20601959 PMC3000544

[B6] Ule J Stefani G Mele A Ruggiu M Wang X Taneri B Gaasterland T Blencowe BJ Darnell RB An RNA map predicting Nova-dependent splicing regulation. Nature 2006 13 580 586 10.1038/nature05304 17065982

[B7] Wang Z Kayikci M Briese M Zarnack K Luscombe NM Rot G Zupan B Curk T Ule J iCLIP predicts the dual splicing effects of TIA-RNA interactions. PLoS Biol 2010 13 e1000530 10.1371/journal.pbio.1000530 21048981 PMC2964331

[B8] Lozzio CB Wigler PW Cytotoxic effects of thiopyrimidines. J Cell Physiol 1971 13 25 32 10.1002/jcp.1040780105 5165085

[B9] Granneman S Kudla G Petfalski E Tollervey D Identification of protein binding sites on U3 snoRNA and pre-rRNA by UV cross-linking and high-throughput analysis of cDNAs. Proc Natl Acad Sci USA 2009 13 9613 9618 10.1073/pnas.0901997106 19482942 PMC2688437

[B10] Kishore S Jaskiewicz L Burger L Hausser J Khorshid M Zavolan M A quantitative analysis of CLIP methods for identifying binding sites of RNA-binding proteins. Nat Methods 2011 13 559 564 10.1038/nmeth.1608 21572407

[B11] Zhang C Darnell RB Mapping in vivo protein-RNA interactions at single-nucleotide resolution from HITS-CLIP data. Nat Biotechnol 2011 13 607 614 10.1038/nbt.1873 21633356 PMC3400429

[B12] Urlaub H Hartmuth K Luhrmann R A two-tracked approach to analyze RNA-protein crosslinking sites in native, nonlabeled small nuclear ribonucleoprotein particles. Methods 2002 13 170 181 10.1016/S1046-2023(02)00020-8 12054894

[B13] Buckanovich RJ Darnell RB The neuronal RNA binding protein Nova-1 recognizes specific RNA targets in vitro and in vivo. Mol Cell Biol 1997 13 3194 3201 9154818 10.1128/mcb.17.6.3194 PMC232172

[B14] Jensen KB Musunuru K Lewis HA Burley SK Darnell RB The tetranucleotide UCAY directs the specific recognition of RNA by the Nova K-homology 3 domain. Proc Natl Acad Sci USA 2000 13 5740 5745 10.1073/pnas.090553997 10811881 PMC18503

[B15] Lewis HA Musunuru K Jensen KB Edo C Chen H Darnell RB Burley SK Sequence-specific RNA binding by a Nova KH domain: implications for paraneoplastic disease and the fragile X syndrome. Cell 2000 13 323 332 10.1016/S0092-8674(00)80668-6 10676814

[B16] Musunuru K Darnell RB Determination and augmentation of RNA sequence specificity of the Nova K-homology domains. Nucleic Acids Res 2004 13 4852 4861 10.1093/nar/gkh799 15367696 PMC519101

[B17] Dredge BK Stefani G Engelhard CC Darnell RB Nova autoregulation reveals dual functions in neuronal splicing. EMBO J 2005 13 1608 1620 10.1038/sj.emboj.7600630 15933722 PMC1142566

[B18] Teplova M Malinina L Darnell JC Song J Lu M Abagyan R Musunuru K Teplov A Burley SK Darnell RB Patel DJ Protein-RNA and protein-protein recognition by dual KH1/2 domains of the neuronal splicing factor Nova-1. Structure 2011 13 930 944 10.1016/j.str.2011.05.002 21742260 PMC3134789

[B19] King MY Redman KL RNA methyltransferases utilize two cysteine residues in the formation of 5-methylcytosine. Biochemistry 2002 13 11218 11225 10.1021/bi026055q 12220187

[B20] Hussain S Benavente SB Nascimento E Dragoni I Kurowski A Gillich A Humphreys P Frye M The nucleolar RNA methyltransferase Misu (NSun2) is required for mitotic spindle stability. J Cell Biol 2009 13 27 40 10.1083/jcb.200810180 19596847 PMC2712989

[B21] Motorin Y Lyko F Helm M 5-methylcytosine in RNA: detection, enzymatic formation and biological functions. Nucleic Acids Res 2010 13 1415 1430 10.1093/nar/gkp1117 20007150 PMC2836557

[B22] Redman KL Assembly of protein-RNA complexes using natural RNA and mutant forms of an RNA cytosine methyltransferase. Biomacromolecules 2006 13 3321 3326 10.1021/bm051012l 17154459

[B23] Tollervey JR Curk T Rogelj B Briese M Cereda M Kayikci M Konig J Hortobagyi T Nishimura AL Zupunski V Patani R Chandran S Rot G Zupan B Shaw CE Ule J Characterizing the RNA targets and position-dependent splicing regulation by TDP-43. Nat Neurosci 2011 13 452 458 10.1038/nn.2778 21358640 PMC3108889

[B24] Forch P Puig O Martinez C Seraphin B Valcarcel J The splicing regulator TIA-1 interacts with U1-C to promote U1 snRNP recruitment to 5' splice sites. EMBO J 2002 13 6882 6892 10.1093/emboj/cdf668 12486009 PMC139089

[B25] Aznarez I Barash Y Shai O He D Zielenski J Tsui LC Parkinson J Frey BJ Rommens JM Blencowe BJ A systematic analysis of intronic sequences downstream of 5' splice sites reveals a widespread role for U-rich motifs and TIA1/TIAL1 proteins in alternative splicing regulation. Genome Res 2008 13 1247 1258 10.1101/gr.073155.107 18456862 PMC2493427

[B26] Zhou Y Cheunsuchon P Nakayama Y Lawlor MW Zhong Y Rice KA Zhang L Zhang X Gordon FE Lidov HG Bronson RT Klibanski A Activation of paternally expressed genes and perinatal death caused by deletion of the Gtl2 gene. Development 2010 13 2643 2652 10.1242/dev.045724 20610486 PMC2910384

[B27] Zhang X Rice K Wang Y Chen W Zhong Y Nakayama Y Zhou Y Klibanski A Maternally expressed gene 3 (MEG3) noncoding ribonucleic acid: isoform structure, expression, and functions. Endocrinology 2010 13 939 947 10.1210/en.2009-0657 20032057 PMC2840681

[B28] Hamburgh ME Curr KA Monaghan M Rao VR Tripathi S Preston BD Sarafianos S Arnold E Darden T Prasad VR Structural determinants of slippage-mediated mutations by human immunodeficiency virus type 1 reverse transcriptase. J Biol Chem 2006 13 7421 7428 10.1074/jbc.M511380200 16423828

[B29] Jelen N Ule J Zivin M Darnell RB Evolution of Nova-dependent splicing regulation in the brain. PLoS Genet 2007 13 1838 1847 17937501 10.1371/journal.pgen.0030173 PMC2014790

[B30] Buratti E Baralle FE Characterization and functional implications of the RNA binding properties of nuclear factor TDP-43, a novel splicing regulator of CFTR exon 9. J Biol Chem 2001 13 36337 36343 10.1074/jbc.M104236200 11470789

[B31] Konig J Zarnack K Luscombe NM Ule J Protein-RNA interactions: new genomic technologies and perspectives. Nat Rev Genet 2011 13 77 83 10.1038/ni.2154 22251872

[B32] Chi SW Zang JB Mele A Darnell RB Argonaute HITS-CLIP decodes microRNA-mRNA interaction maps. Nature 2009 13 479 486 19536157 10.1038/nature08170 PMC2733940

[B33] Zhang C Frias MA Mele A Ruggiu M Eom T Marney CB Wang H Licatalosi DD Fak JJ Darnell RB Integrative modeling defines the Nova splicing-regulatory network and its combinatorial controls. Science 2010 13 439 443 10.1126/science.1191150 20558669 PMC3412410

[B34] Langmead B Trapnell C Pop M Salzberg SL Ultrafast and memory-efficient alignment of short DNA sequences to the human genome. Genome Biol 2009 13 R25 10.1186/gb-2009-10-3-r25 19261174 PMC2690996

[B35] Novoalign http://www.novocraft.com/

[B36] iCount http://icount.biolab.si

[B37] Crooks GE Hon G Chandonia JM Brenner SE WebLogo: a sequence logo generator. Genome Res 2004 13 1188 1190 10.1101/gr.849004 15173120 PMC419797

[B38] Fejes AP Robertson G Bilenky M Varhol R Bainbridge M Jones SJ FindPeaks 3.1: a tool for identifying areas of enrichment from massively parallel short-read sequencing technology. Bioinformatics 2008 13 1729 1730 10.1093/bioinformatics/btn305 18599518 PMC2638869

