# Supplementary material for: Analysis of CLIP and iCLIP methods for nucleotide-resolution studies of protein-RNA interactions
Source: Genome Biol. 2012 Aug 3;13(8):R67. doi: 10.1186/gb-2012-13-8-r67 (PMC4053741; doi:10.1186/gb-2012-13-8-r67)
Supplement: Additional file 1 — Supplementary Figures s1 to s12. [file gb-2012-13-8-r67-S1.PDF]

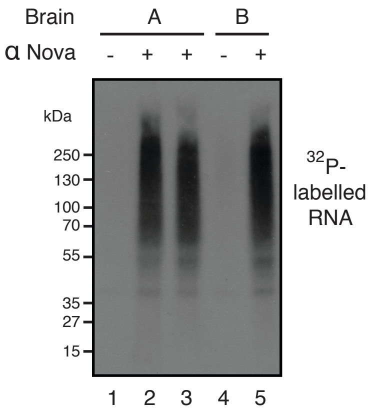

Figure s1 Autoradiograph of Nova iCLIP experiment. Cross-linked Nova-RNA complexes purified with an antibody against the Nova protein were analysed using denaturing gel electrophoresis and western blotting. Visualization of the complexes was achieved through radioactive labelling of the 5' ends of the RNA with ^{32}P. Nova-RNA complexes can be observed shifting upwards from the molecular weight of the protein (±50 kDa, lanes 2,3 and 5). No signal was observed when no antibody was used during the purification (lanes 1 and 4). Protein extracts were prepared from two mouse brains A and B, that where harvested on postnatal day 0 and 1, respectively (lanes 1-3 and 4,5, respectively).

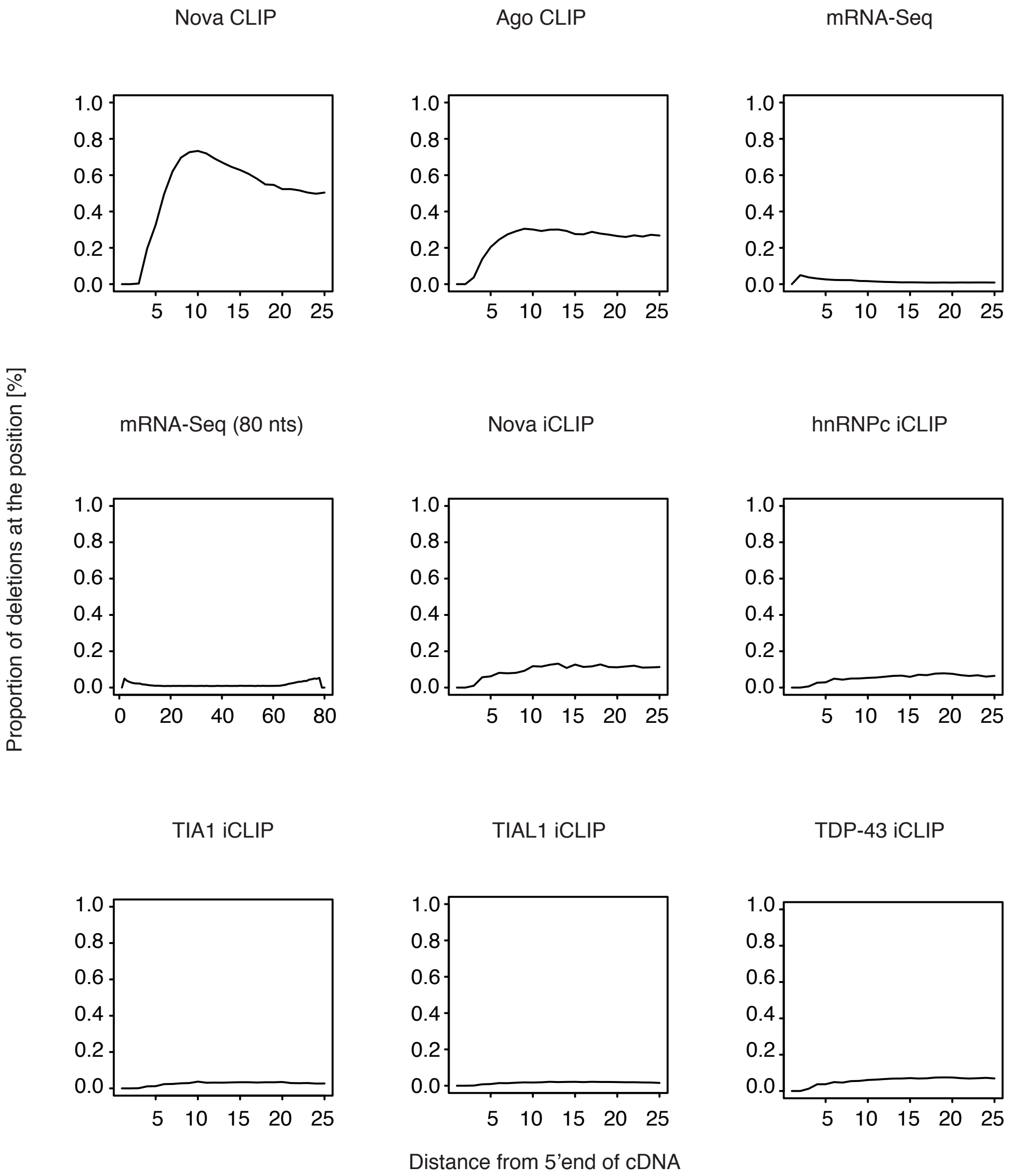

Figure s2 Distribution of deletions in CLIP, mRNA-Seq and iCLIP cDNAs. The proportion of deletions at a given distance from the 3' end of the cDNAs. The protein and the type of experiment are shown on top of each panel. For all experiments, the proportion from position 1 to 25 was plotted, while for mRNA-Seq the proportion from position 1 to 80 was also plotted due to longer length of cDNAs and sequence reads.

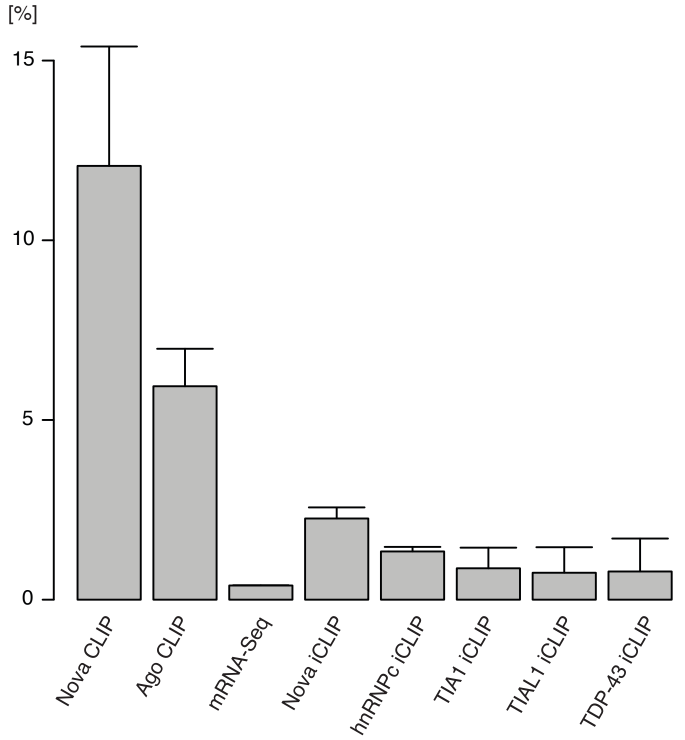

Figure s3 The average ratio of cDNAs with deletions in the first 25 nucleotides of each experiment. Error bar indicates the standard deviations. Nova CLIP: n = 23, Ago CLIP: n = 5, mRNA-Seq: n = 3, Nova iCLIP: n = 3, hnRNPc iCLIP: n = 3, TIA1 iCLIP: n = 6, TIAL1 iCLIP: n = 9 and TDP-43 iCLIP: n = 4.

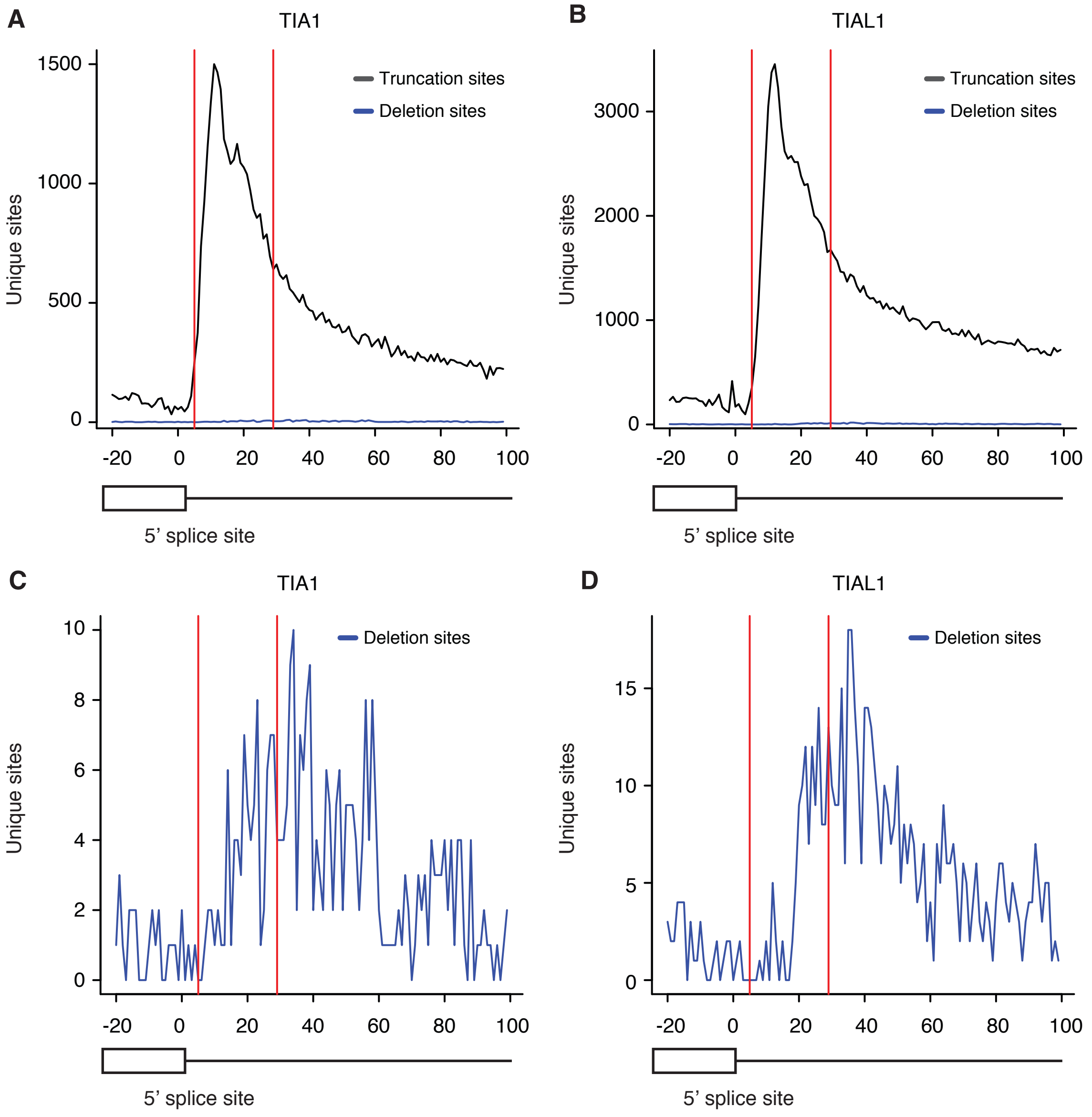

Figure s4 TIA1 and TIAL1 RNA map around 5’splice site exon-intron junction.
(A) The occurrence of TIA1 iCLIP cDNA truncations or deletions around 5’splice site exon-intron junction was plotted. The area (from +6 to +30 downstream of exon-intron junctions), which was used for the comparison of truncations and deletions, was surrounded by red lines. **(B)** Similar to **A**, but that of TIAL1 iCLIP cDNA truncations. **(C)** Similar to **A**, but different Y axis scale. **(D)** Similar to **B**, but different Y axis scale.

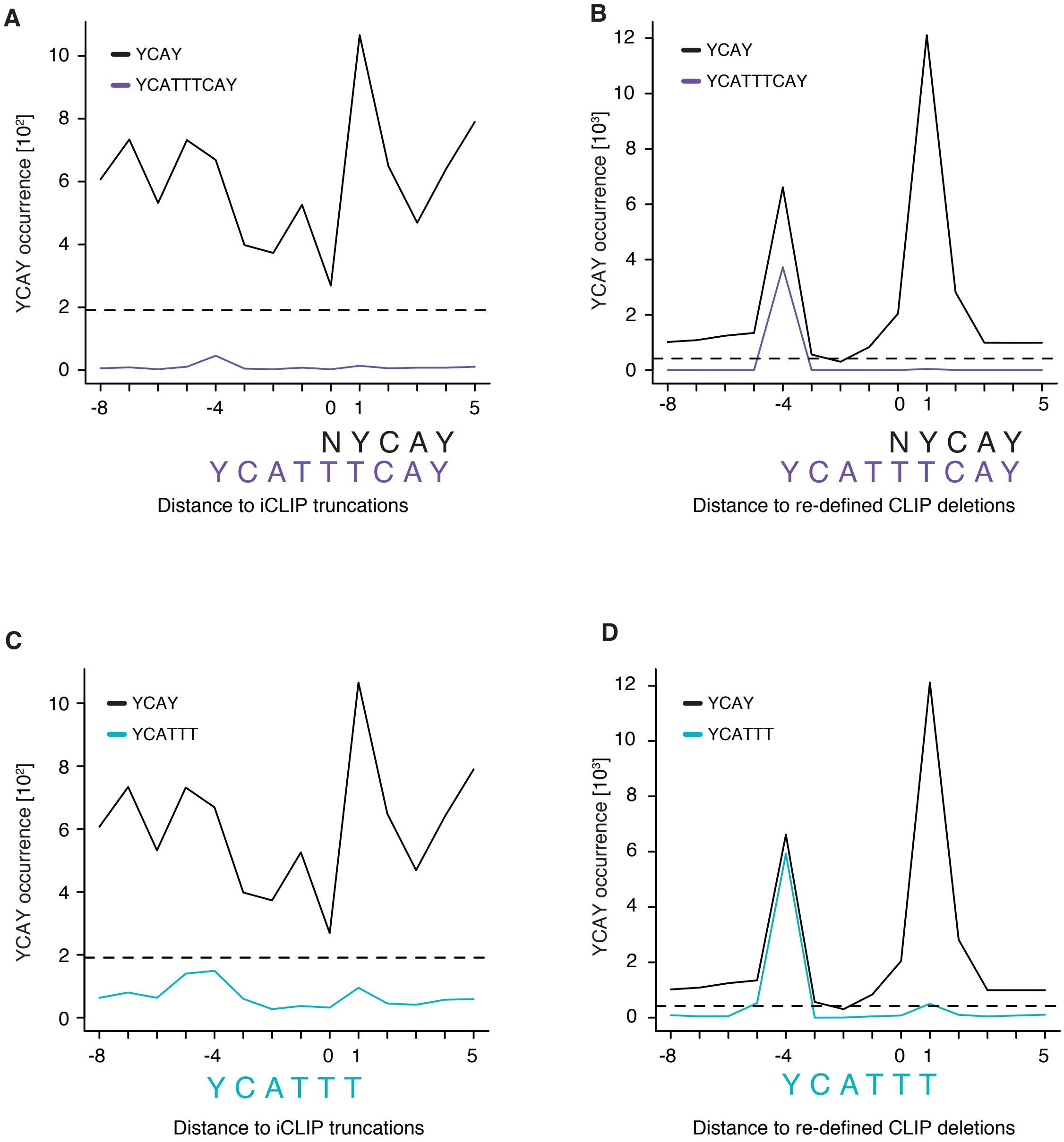

Figure s5 Occurrence of YCAY motif and its variants around CLIP cDNA deletions and iCLIP cDNA truncations. (A) The occurrence of YCAY motifs relative to iCLIP cDNA truncation sites (FDR < 0.05). The starting position of YCAY (black line) or YCATTTCAY motif (purple line) is plotted. Dashed line shows the background occurrence of YCAY motifs. **(B)** Similar to **A**, but showing the occurrence relative to re-defined CLIP cDNA deletion sites (FDR < 0.001). **(C)** Similar to **A**, but showing the starting position of YCAY (black line) or YCATTT motif (light blue line). **(D)** Similar to **C**, but showing the occurrence relative to re-defined CLIP cDNA deletion sites (FDR < 0.001).

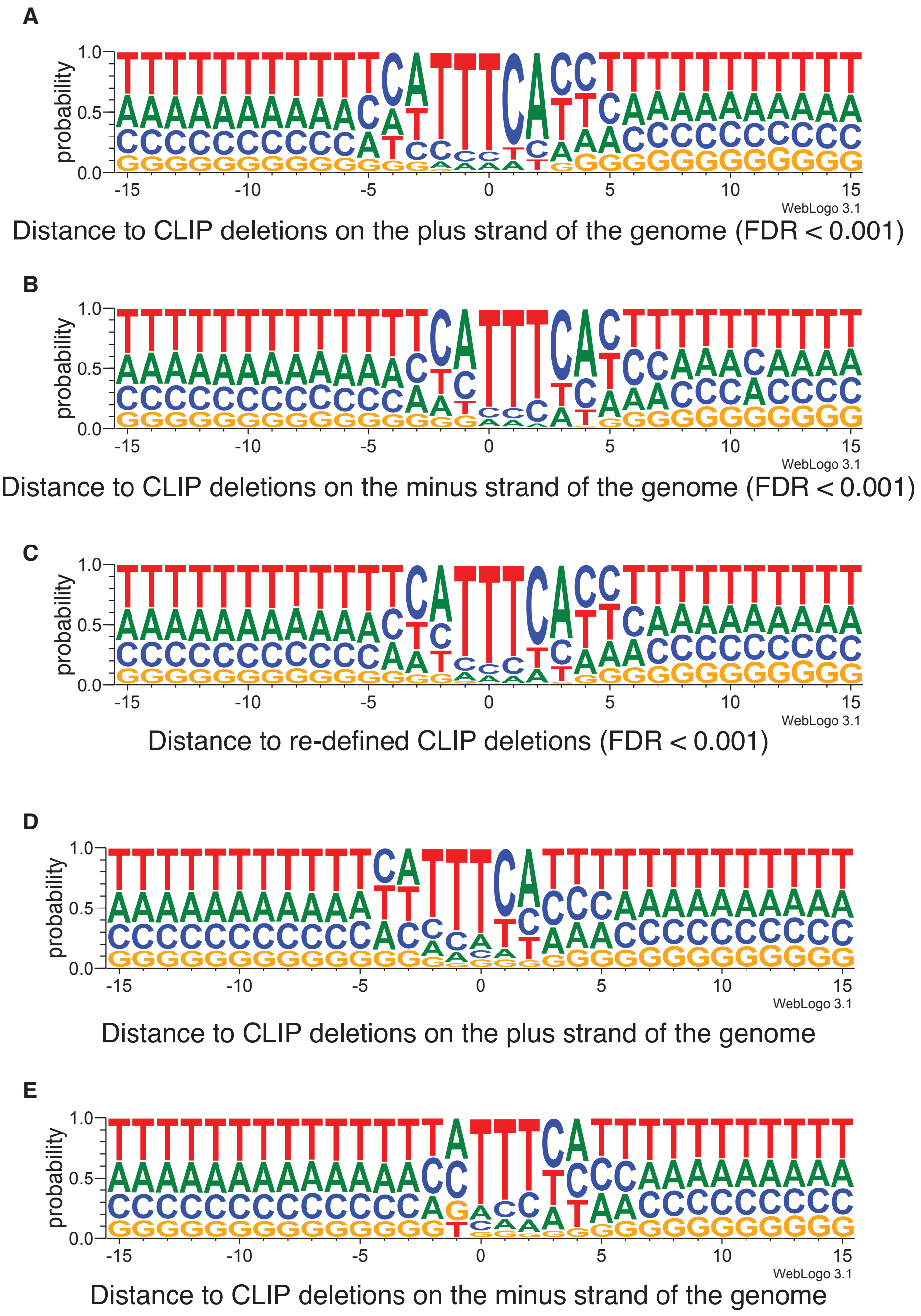

Figure s6 Nucleotide composition around Nova CLIP cDNA deletion sites. (A) The nucleotide compositions around confident Nova CLIP cDNA deletion sites (FDR < 0.001) on the plus strand of the genome. **(B)** Similar to **A**, but confident deletion sites on the minus strand of the genome. **(C)** Similar to **A**, but around confident re-defined Nova CLIP cDNA deletion sites on both strands of the genome. **(D)** Similar to **A**, but around all Nova CLIP cDNA deletion sites (without FDR threshold) on the plus strand of the genome. **(E)** Similar to **D**, but around all Nova CLIP cDNA deletion sites on the minus strand of the genome.

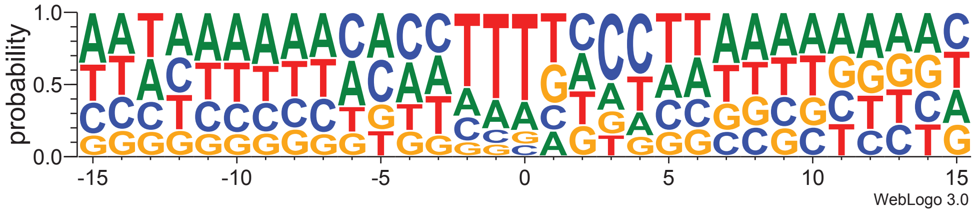

Distance to CLIP deletions on the plus strand of the genome (FDR < 0.001)

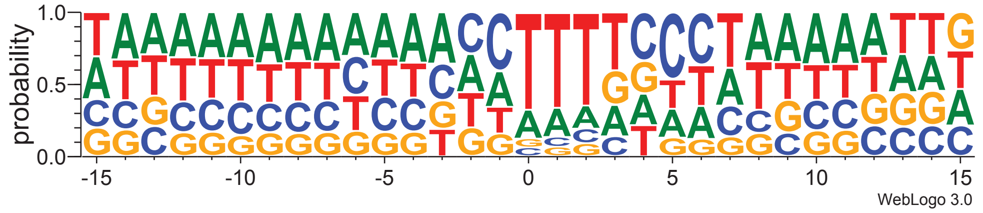

Distance to CLIP deletions on the minus strand of the genome (FDR < 0.001)

Figure s7 Nucleotide compositions around Ago CLIP cDNA deletion sites. The top panel shows the nucleotide compositions around Ago CLIP cDNA deletion sites (FDR < 0.001) on the plus strand of the genome and the bottom panel shows that on the minus strand.

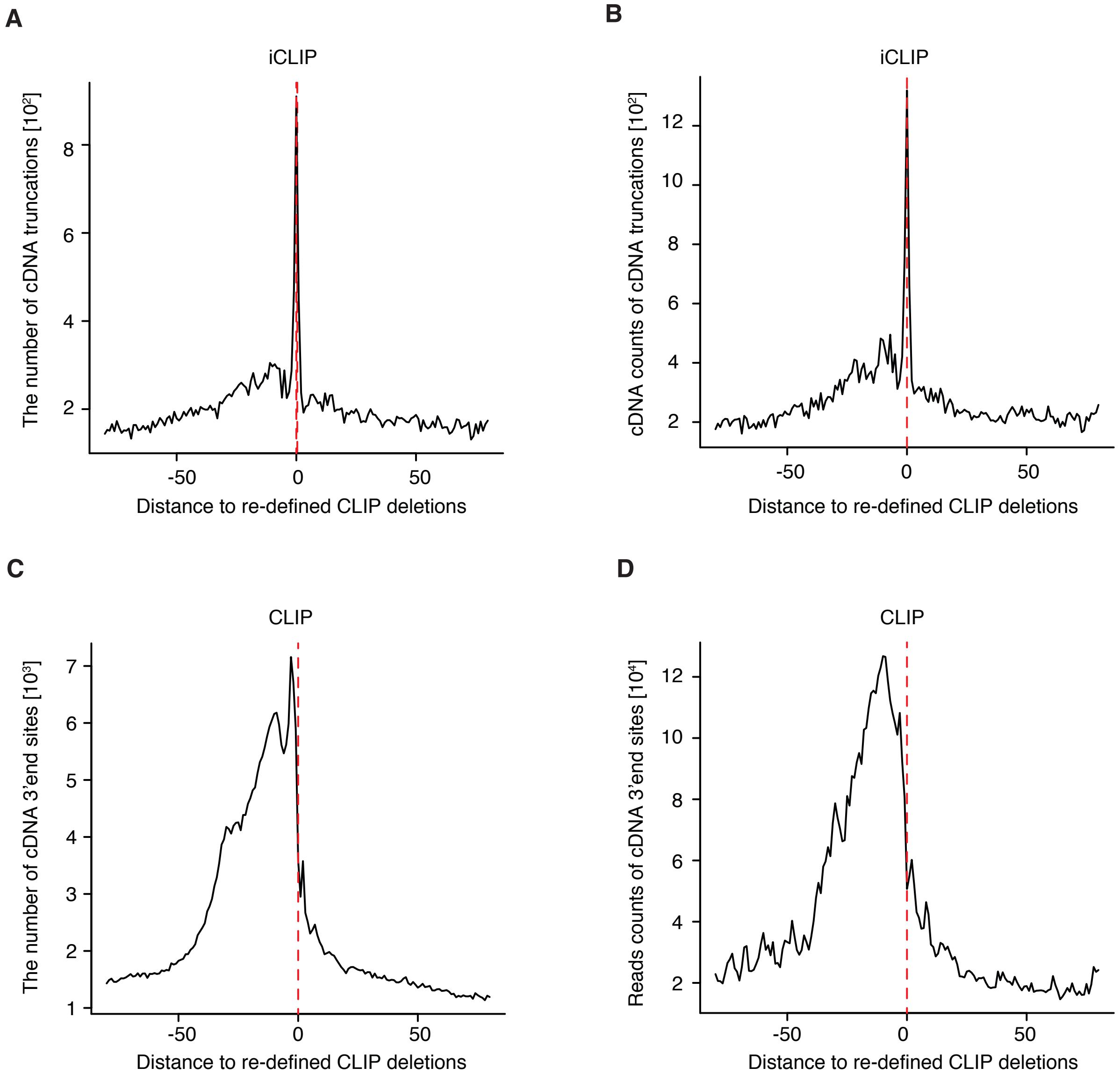

Figure s8 iCLIP and CLIP cDNA truncation sites around CLIP re-defined deletion sites. (A) Occurrence of iCLIP cDNA truncations within 80 nucleotide of the re-defined CLIP deletions. **(B)** Similar to **A**, but the sites were weighted by the number of cDNAs truncated at each site (cDNA counts were determined by considering the random barcode). This panel is the same as in Fig. 2d, but here it serves for comparison with the other panels. **(C)** Similar to **A**, but showing 3'end sites of CLIP cDNAs that lack deletions. For proper comparison with iCLIP truncation sites, we used Nova CLIP cDNAs without deletions from postnatal mouse brain tissues, instead of using all libraries. **(D)** Similar to **C**, but the sites were weighted by the number of reads ended at each site. Since CLIP cDNAs did not have random barcode, we used the total number of reads.

E

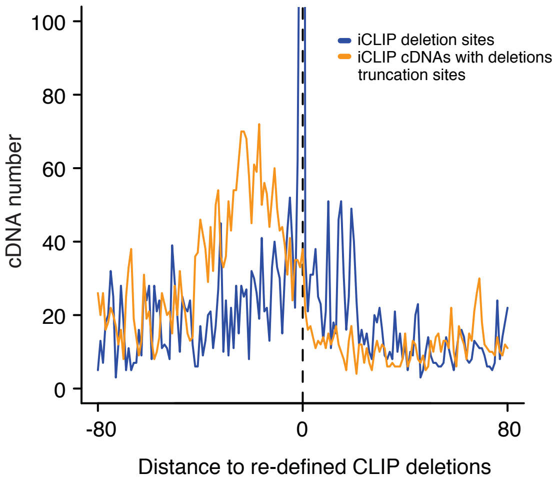

Figure s8 (Continued) **(E)** Enlarged version of Figure 2d's small panel. Occurrence of iCLIP cDNA truncations (orange) or deletions (blue) within 80 nucleotides of the re-defined CLIP deletion sites. iCLIP cDNA deletion sites were re-defined as described in Methods.

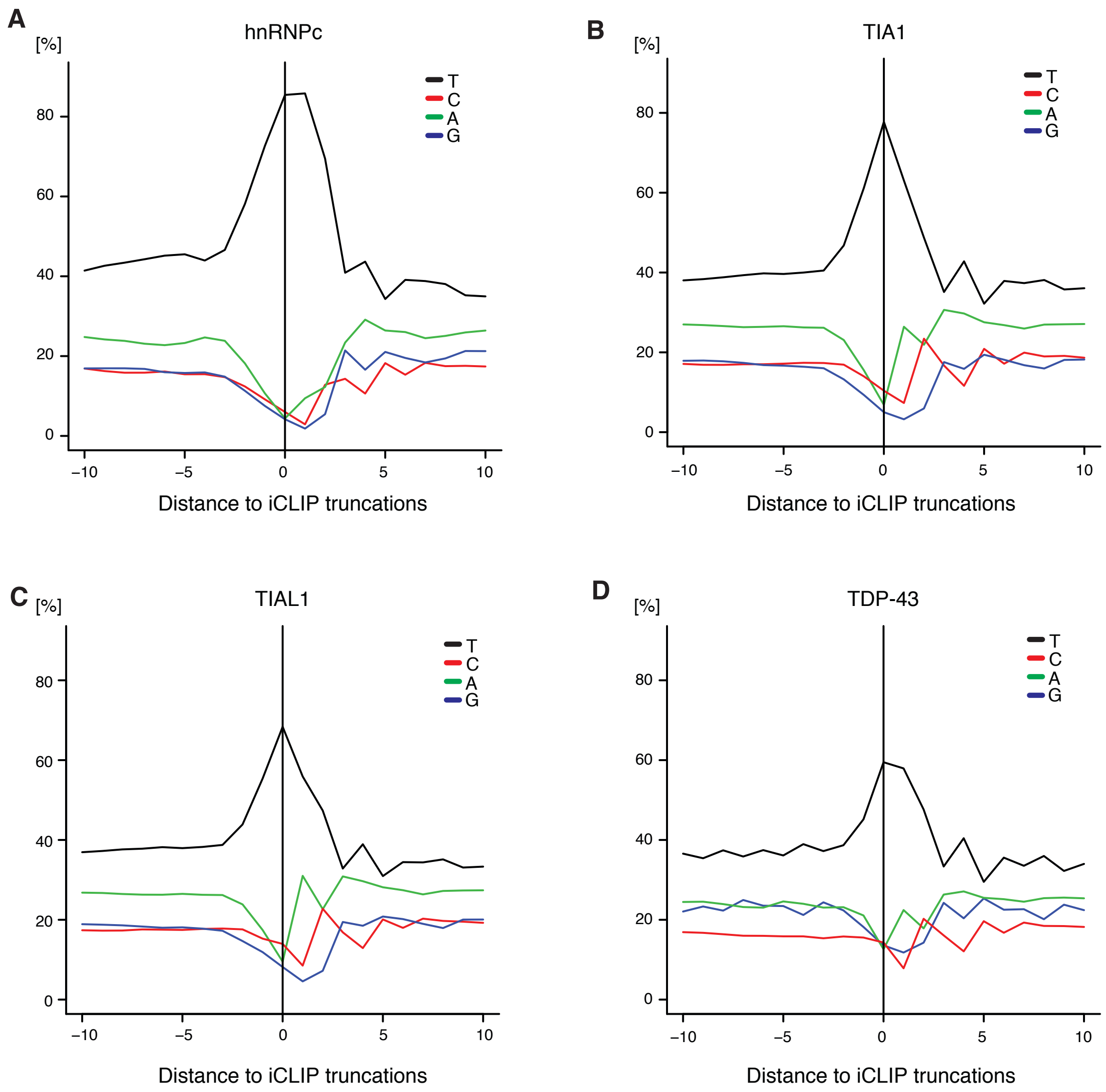

Figure s9 Nucleotide compositions around cross-link sites identified by iCLIP cDNA truncations in protein-coding genes (including introns). The target proteins are shown on top of each panel.

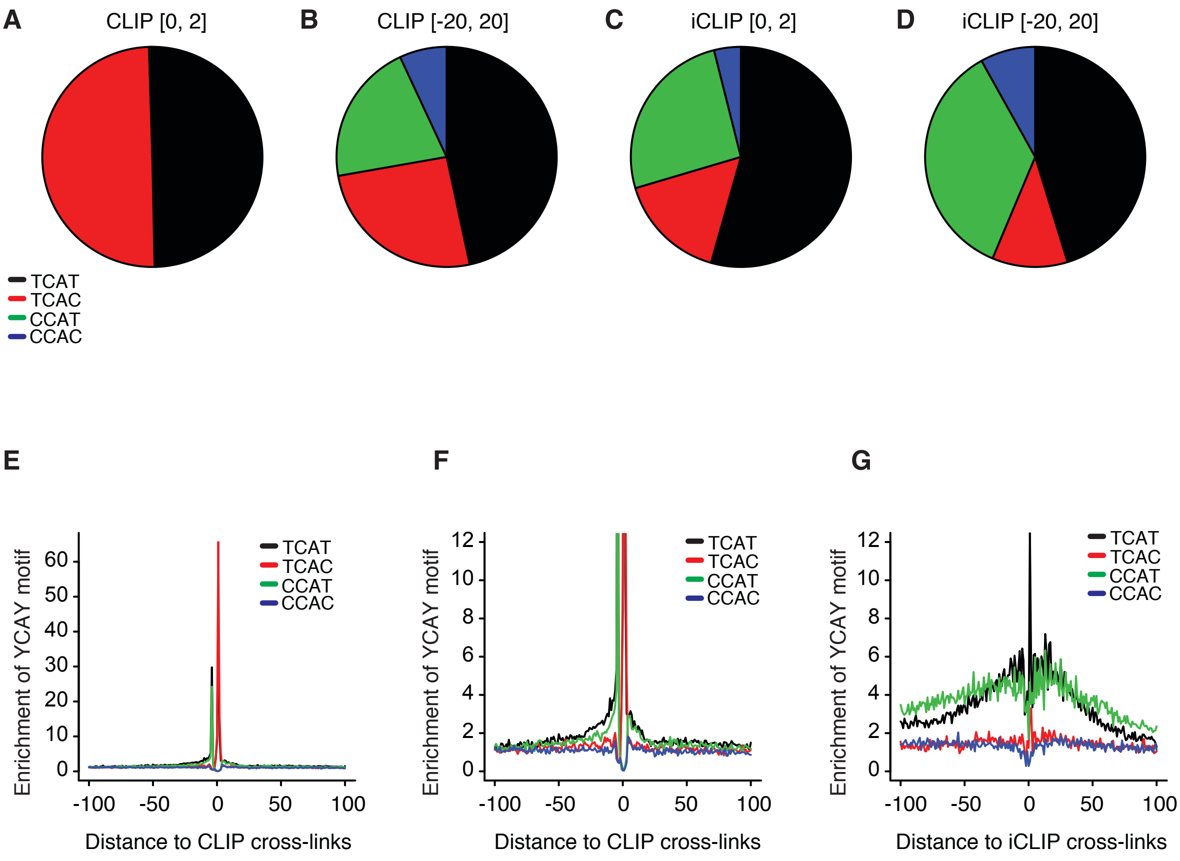

Figure s10 The proportion of 4 types of YCAY motif around Nova CLIP and iCLIP identified low FDR cross-link sites (FDR < 0.001 and FDR < 0.05 each). (A-D) The analyzed area and experiment type was shown on top of each panel. **(E)** The enrichment of each type of YCAY motif around CLIP identified cross-link sites. **(F)** Similar to **E**, but the same scale of enrichment as that of iCLIP shown in **G**. **(G)** Similar to **E**, but around iCLIP identified cross-link sites.

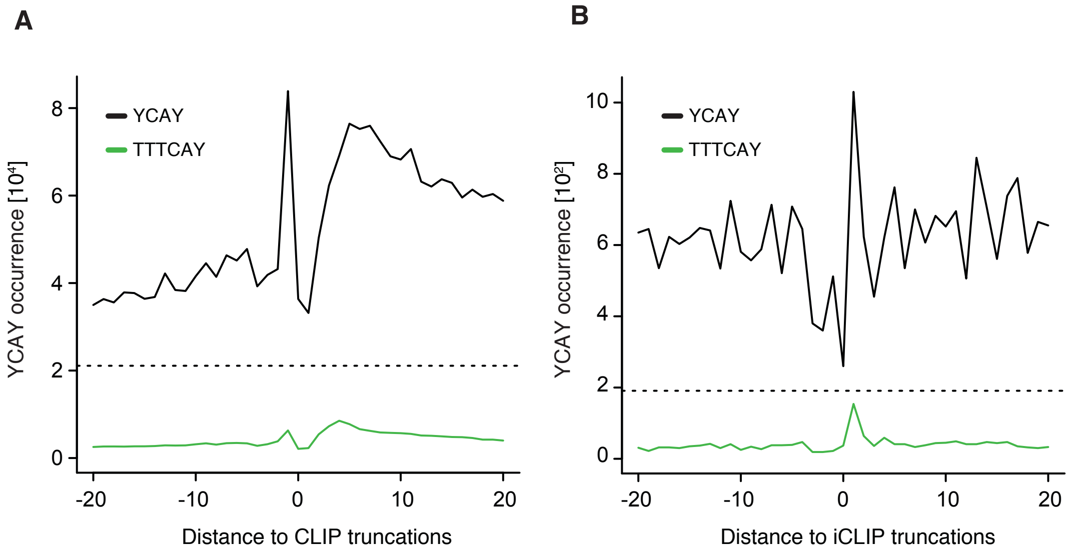

Figure s11 Occurrence of YCAY and TTTCAY motif around clustered iCLIP and CLIP cDNAs truncation sites without deletions. (A) The occurrence of YCAY motifs relative to clustered CLIP cDNA truncation sites (FDR < 0.05). The starting position of YCAY (black line) or YCAY motif following TT and starting T (TTTCAY) (light green line) is plotted. Dashed line shows the background occurrence of YCAY motifs. The position +1 corresponds to the 3'end of CLIP cDNAs. The peak at position -1 is likely to be due to the preference of RNase A, which cleaves only after C and U nucleotides. Unlike those around iCLIP cDNA truncation sites, the enrichment of YCAY motif is asymmetric around CLIP cDNA truncation sites, indicating that these cDNAs read through cross-link sites. **(B)** Similar to **A**, but around iCLIP cDNA truncation sites.

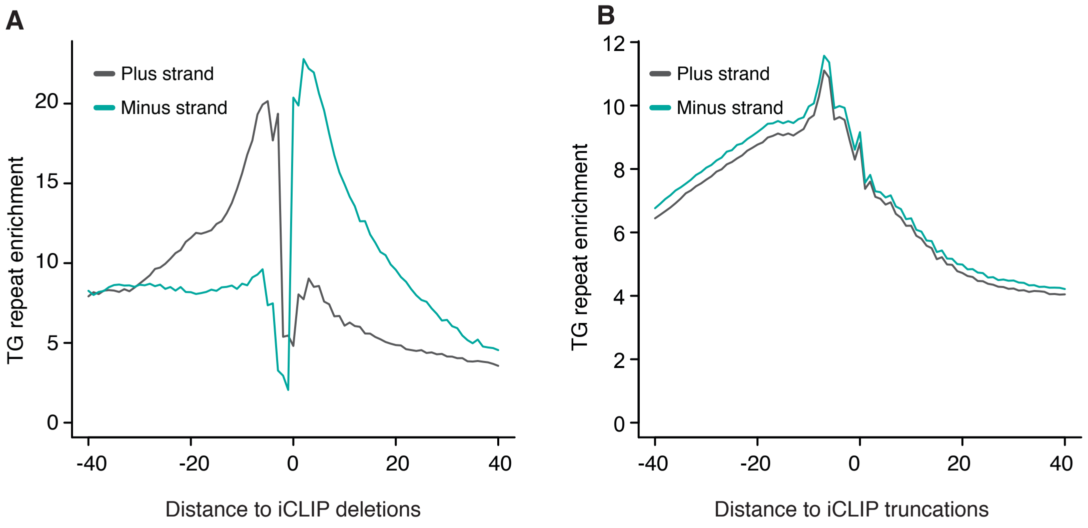

Figure s12 Comparison of TG repeat enrichment around iCLIP cDNA deletions and iCLIP cDNA truncations. (A) TG repeat enrichment around TDP-43 iCLIP cDNA deletions. TG repeat enrichment was defined as the enrichment of two 5-mers, "TGTGT" and "GTGTG", such that the starting position of each motif is evaluated. **(B)** Similar to **A**, but TG repeat enrichment around iCLIP cDNA truncations is evaluated.

Our interpretation of this result is as follows: The distribution of TG repeat around iCLIP cDNA truncations is similar on both strands of the genome, as expected. However, the distribution of TG repeats enrichment around the CLIP cDNA deletions on the plus strand is symmetrical to that of the minus strand. Similar to the T deletions in the TTT motifs at Nova CLIP cDNA deletions, this symmetrical distribution indicates an inability of the mapping software to precisely define the position of the TDP-43 iCLIP cDNA deletion sites. However, if single nucleotide was deleted from a TG repeat, it would be possible to identify the exact position of deletion sites. Therefore, we speculate that most deletion sites in TG repeats correspond to dinucleotide deletions. Since dinucleotide deletions in TG repeats are a common type of the genome variation, this exemplifies the difficulty in distinguishing cDNA deletions that result from cross-link-induced mutations from those that result from genome variation.
